# Invasive Disease and Toxic Shock due to Zoonotic Streptococcus suis: An Emerging Infection in the East?

**DOI:** 10.1371/journal.pmed.0030187

**Published:** 2006-04-11

**Authors:** Shiranee Sriskandan, Josh D Slater

## Abstract

Sriskandan and Slater discuss the implications of Tang and colleagues' report of the largest known zoonotic outbreak of *S. suis,* which occurred in Sichuan Province, China, in 2005.

Streptococcus suis ( S. suis) is widely recognised in the veterinary world as a cause of rapidly progressive and fatal sepsis in infant pigs, associated with meningitis, polyarthritis, and, occasionally, pneumonia. A more fulminant bacteraemic infection is seen in neonatal pigs [ 1, 2]. For reasons that are unknown, adult pigs do not succumb to this infection, although they demonstrate asymptomatic nasopharyngeal carriage ( Figure 1), and infant piglets become infected after early contact with colonised adult females. In many respects, S. suis infection is the porcine version of human group B strep disease. Zoonotic disease due to S. suis does occur sporadically in Western countries, but is encountered more commonly in countries such as Thailand, China, and Hong Kong. It affects predominantly individuals with occupational exposure to pigs. In humans, meningitis is the most common presentation (about 80% of all cases), though S. suis disease can be associated with other suppurative complications such as pneumonia and arthritis. Mortalities of 5%–10% of cases are reported [ 2], and S. suis is the third most common cause of bacterial meningitis in Hong Kong [ 3].

**Figure 1:**
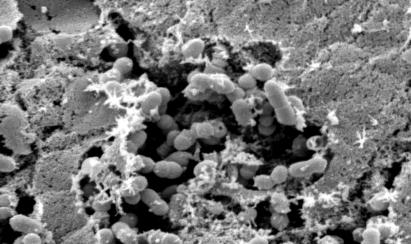
Scanning Electron Microscopy of S. suis Diplococci in a Sheet of Mucus on the Surface of the Nasopharyngeal Epithelium from a Pig (Photo: Josh D. Slater)

## S. suis and Toxic Shock

In the current issue of *PLoS Medicine,* Tang and colleagues report on the largest known zoonotic outbreak of S. suis, which occurred in Sichuan Province in China in 2005 [ 4]. The outbreak, which attracted considerable public and scientific interest [ 5], killed 38 out of 204 individuals with the infection and coincided with a major outbreak of disease in pigs. In addition to its size and the associated high mortality, this outbreak is unique in that a large proportion of patients were victim to a toxic shock-like syndrome (TSLS). Indeed, the vast majority of deaths in this outbreak occurred in patients with TSLS rather than in patients with meningitis. To date, streptococcal toxic shock, per consensus definition, has been limited to disease caused by the group A streptococcus, S. pyogenes [ 6]. Sporadic reports of TSLS due to other (nongroup A) beta-haemolytic streptococci have been reported, though there is potentially considerable diagnostic overlap between cases of septic shock and TSLS [ 7]. With one exception [ 8], S. suis has not previously been linked to TSLS. Tang and colleagues report that almost all human cases of TSLS due to S. suis demonstrated an erythematous blanching rash, one of the most specific features of TSLS, associated with hypotension and multiorgan failure. Tang and colleagues provide insight into the clinical outbreak with regard to the spectrum of clinical disease, morbidity, and mortality. They did not identify a clear focus of infection in the TSLS cases, in contrast to the originally described streptococcal toxic shock syndrome, where soft tissue infection was most commonly found.

## S. suis—The Source?

Epidemiologically, all cases of human disease in the recent outbreak were linked to exposure to pigs, and, although broken skin is reported in most cases, the precise route of transmission is not further discussed. Other sporadic zoonotic S. suis cases have been linked to accidental inoculation injuries through lacerations or inhalation of infected aerosols from infected carcasses, though S. suis is also found in faeces and fomites of infected herds [ 1, 2]. At least some of the human cases in an earlier S. suis outbreak in Sichuan Province in 1998 may have been related to ingestion of contaminated food [ 9]. Farmers in Sichuan Province have close contact with their pigs; humans and animals often share the same accommodations and animals are slaughtered at home. Furthermore, it is common practice for diseased animals to be slaughtered and eaten. Thus, in addition to inoculation and airborne transmission from infected pigs, foodborne transmission by infected pork may have also contributed to the recent outbreak. [Fig pmed-0030187-g001]

**Figure pmed-0030187-g001:**
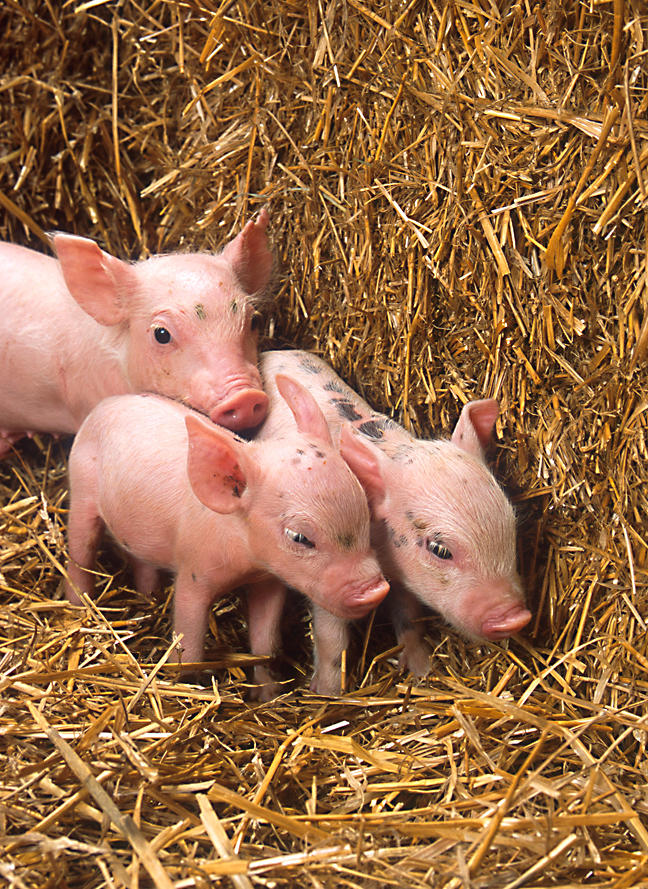
S. suis causes a rapidly progressive and fatal sepsis in infant pigs

## Phenotype of Virulent S. suis Isolates: Lack of Superantigenicity?

Having identified S. suis as the causative agent of the 2005 outbreak, the authors went on to provide an initial phenotypic and molecular analysis of the S. suis strains involved in clinical disease. Bacterial superantigen production, with consequent overstimulation of immunological cascades, is believed to underlie the catastrophic features of toxic shock [ 10]. However, little is known about the superantigenicity of S. suis, and when Tang and colleagues tested S. suis isolates for mitogenic activity, a hallmark of superantigen production, they found none. In the absence of superantigens, alternative virulence mechanisms might account for endothelial leakage and the features of TSLS, such as M protein–fibrinogen interactions and activation of the coagulation system [ 10, 11]. More detailed analysis of the strains associated with the outbreak is required to eliminate a role for superantigens; this includes molecular analysis and study of T cell subset changes induced by exposure to secreted products of these strains. The genome sequences of two S. suis strains are currently available for study (see http://www.sanger.ac.uk/Projects/S_suis and http://genome.jgi-psf.org/draft_microbes/strsu/strsu.home.html), and although regions bearing some similarity to S. pyogenes M protein and superantigen genes can be identified, functional analysis is needed to fully characterise these features.

## In Vivo Study of S. suis Virulence

One of the key questions arising from this report is whether a new, highly virulent strain of S. suis has emerged in China? Tang and colleagues explored virulence using a neonatal piglet model of infection. Consistent with its causative role, the S. suis strain from the recent outbreak caused disease, whereas a known avirulent S. suis strain did not. Future studies might be best served by a comparison of invasive isolates from the current outbreak with those causing invasive disease in other countries, in addition to a comparison with colonising isolates. Being the natural host, the pig is clearly a superior model for study of S. suis, and mouse models have proved unreliable [ 12]. Future refinement and standardisation of S. suis models should include the use of pathogen-free piglets and the administration of an inoculum via the nasopharyngeal route, in order to better reproduce natural infection. Pigs are normally sensitive to the effects of superantigens [ 13], although they have not been reported to develop the rash characteristic of human TSLS.

## Comparison of Chinese Outbreak Isolates with Other Virulent Strains

To compare the S. suis isolates of the Chinese outbreak with European invasive isolates, molecular analysis was undertaken by the investigating team. S. suis is an alpha-haemolytic, or nonhaemolytic, group D streptococcus, which is normally sensitive to penicillins in vitro. Strains associated with virulent disease do produce a number of extracellular proteins, such as a 110-kDa extracellular factor, a 136-kDa muramidase-released protein, and a thiol-activated haemolysin (suilysin), although none alone are sufficient for full virulence [ 14]. The capsular polysaccharide, as for other streptococci, appears crucial to resistance to opsonophagocytosis [ 15]. There are 35 known capsular serotypes, though capsular serotype 2—the type associated with the 2005 outbreak in China—is the most common in most parts of the world. Even within this serotype, there is heterogeneity, and European strains appear more likely to produce virulence-associated exoproteins than North American strains [ 14]. Isolates from the 2005 outbreak in Sichuan Province were compared at a molecular level with a European serotype-2 strain (P1/7) and with a handful of isolates from the earlier outbreak in Sichuan Province in 1998.

The isolates from the recent Chinese outbreak carried genes for all of the known virulence-associated factors, akin to European invasive strains. However, Tang and colleagues report that the serotype-2 strain associated with the recent outbreak is quite different to other invasive type-2 isolates with regard to the capsular polysaccharide synthesis gene cluster and restriction fragment length polymorphism pattern. Intriguingly, the recent isolates are indistinguishable from the isolates that caused the earlier 1998 outbreak in China that killed 14 out of 25 human patients [ 9]. Thus, there appears to be good evidence that the strains from the Chinese outbreaks are epidemiologically distinct from other invasive S. suis strains.

## Emerging S. suis Infections in Humans: A Need for Coordinated and Cooperative Research

S. suis infection is of major economic and veterinary importance in the farming world, and should now be on the list of differential diagnoses when clinicians encounter patients with unexplained sepsis who have a history of exposure to pigs. Eradication of S. suis from the pig population is not currently feasible, even though S. suis appears fully sensitive to penicillin. Antibiotic treatment in pigs is rarely successful, probably because of poor antibiotic penetration of the porcine tonsillar tissues, which act as a source of infection [ 16]. Resistance to macrolides, lincosamides, and tetracycline is common, limiting efforts to clear tonsillar carriage. Development of a vaccine targeted against the most virulent or prevalent strains might be one possible way to prevent colonisation of female pigs and to protect those working with pigs [ 17]. Better characterisation of activities that increase risk of S. suis acquisition and faster diagnostic tests to identify outbreaks in pigs will lead to adaptation of practices that incur unnecessary exposure to infected carcasses.

The emergence of any new zoonotic infectious disease associated with high mortality is of global concern, and there is an urgent need for better characterisation of the S. suis strains involved. Whether the Chinese strain has enhanced virulence per se is at present unclear; the increased mortality seen with the two Chinese outbreaks may simply reflect regional differences in mode of acquisition or in access to treatment. International collaboration between laboratories experienced in virulence analysis and molecular epidemiology is required in order to clarify differences between isolates circulating in different regions of the world.

## Abbreviation

TSLS: toxic shock-like syndrome
